# Identification of immunomodulating properties of postbiotics from lactobacilli using the zebrafish (*Danio rerio*) model

**DOI:** 10.1186/s12917-025-05159-z

**Published:** 2025-11-28

**Authors:** Chamilani Nikapitiya, Jayasinghage Nirmani Chathurangika Jayasinghe, Mawalle Kankanamge Hasitha Madhawa Dias, E. H.T. Thulshan Jayathilaka, Shan Lakmal Edirisinghe, Cheol- Hee Kim, Marion Schiavone, Eric Leclercq, Emmanuelle Apper, Amélie Mugnier, Mahanama De Zoysa

**Affiliations:** 1 College of Veterinary Medicine and Research Institute of Veterinary Medicine, Chungnam National University, Yuseong-gu, Daejeon, 34134 Republic of Korea; 2 Department of Biology, Chungnam National University, Yuseong-gu, Daejeon, 34134 Republic of Korea; 3 Lallemand SAS, Blagnac, 31700 France

**Keywords:** Immunomodulation, Antiviral activity, Postbiotic, Lactobacilli, Zebrafish

## Abstract

**Background & objectives:**

Probiotics are increasingly used in the pet industry to enhance the health and well-being of companion animals. Among them, *Lactobacillus* strains and their metabolites have demonstrated the ability to maintain immune homeostasis, modulate immune responses, and exhibit antiviral properties. Despite growing interest in postbiotics, non-viable microbial products or metabolic byproducts, scientific literature on their effects remains limited. This study investigates the immunomodulatory and antiviral properties of three postbiotics derived from heat-inactivated *Lactobacillus* strains using an adult zebrafish (*Danio rerio)* model challenged with viral hemorrhagic septicemia virus (VHSV).

**Methods:**

A total of 330 zebrafish were assigned to five groups: a non-challenged control (C1), a VHSV-challenged control (C2), and three experimental groups supplemented with one of three heat-treated *Lactobacillus* strains at the same dosage (*Lacticaseibacillus paracasei* HA-108; *Lactiplantibacillus plantarum* HA-119 or *Lactobacillus helveticus* HA-122). Fish were fed at 4% of biomass for 21 days. Following this period, a subset of the fish was used for immune gene expression profiling and histological examination of the gut and kidney. The remaining fish were challenged with VHSV and monitored for survival over 10 days.

**Results:**

All postbiotics treatments modulated immune responses, with *L. plantarum* HA-119 showing the most pronounced effects, including upregulation of key immune genes such as *Il1β* and *Ifn-γ*, indicative of anti-inflammatory and antiviral activity. Histological analysis revealed no significant changes in goblet cell density or villi height, supporting the safety of the postbiotics. Survival rates were significantly higher in the *L. plantarum* HA-119 and *L. helveticus* HA-122 groups compared to the VHSV control.

**Conclusions:**

These findings, derived from a well-established zebrafish model, suggest that postbiotics from *Lactobacillus* strains may enhance antiviral immunity and overall health in vertebrates, supporting their potential as safe, effective microbial-based nutritional interventions in pet nutrition.

**Supplementary Information:**

The online version contains supplementary material available at 10.1186/s12917-025-05159-z.

## Introduction

The health and longevity of companion animals are increasingly prioritized by pet owners, driving demand for nutritional strategies that support immune resilience and overall well-being. Functional ingredients, such as probiotics, have gained attention for their ability to modulate immune responses and promote gut health in dogs and cats. However, the practical application of probiotics in pet nutrition faces significant challenges, including ensuring microbial viability during processing, maintaining stability throughout shelf life, and navigating complex regulatory frameworks.

To address these limitations, postbiotics - recently defined as “a preparation of inanimate microorganisms and/or their components that confers a health benefit on the host” [1] - have emerged as promising alternatives. Unlike probiotics, postbiotics offer enhanced stability and safety, and their bioactive components can exert immunomodulatory, anti-inflammatory, and antiviral effects. Recent studies have demonstrated that heat-inactivated *Lactobacillus* strains can modulate immune responses and confer antiviral protection in vertebrate models, including mice and zebrafish [2–4]. For example, *L. plantarum* and *L. casei* postbiotics have been shown to improve survival after viral challenge by modulating cytokine responses and reducing inflammation. Surface layer proteins from *L. helveticus* have been shown to inhibit pathogen adhesion and attenuate inflammation [5–7].

Although evidence in companion animals is limited, inactivated *Lactobacillus* strains have been reported to modulate microbiota and metabolic biomarkers in dogs [8] and improve disease resistance in fish [9]. In vitro studies further suggest that postbiotics can modulate lactate and short-chain fatty acid production in canine models [10]. Despite these promising findings, scientific validation of postbiotics, particularly at the strain level, remains limited, and their mechanisms of action in vertebrate models relevant to companion animal health are not fully understood.

Given the ethical and regulatory push to reduce the use of mammals in research, particularly dogs and cats, alternative models aligned with the 3Rs principle (Replacement, Reduction, and Refinement) are increasingly adopted. The zebrafish (*Danio rerio*) has emerged as a valuable vertebrate model in both human and veterinary research [11], including proof-of-concept studies for postbiotics [12]. Zebrafish are considered to have a lower level of sentience compared to mammalian models, making their use more ethically acceptable and consistent with the 3Rs framework. Moreover, the zebrafish boasts a sophisticated immune system, encompassing both innate and adaptive immune responses, which closely resemble those of mammals. Consequently, many of the signalling pathways and immune molecules found in mammals are also present and functionally conserved in zebrafish [11]. Overall, zebrafish studies can provide mechanistic insights with translational relevance for companion animals.

Interestingly, several recent reviews have synthesized the growing body of evidence supporting the use of postbiotics in aquaculture. They have shown potential to enhance growth performance, disease resistance, and immune modulation in aquatic species, thereby contributing to more sustainable aquaculture practices [13]– [14]. In addition, Ang et al. [15] further detail the diversity of postbiotic compounds evaluated in aquaculture species, including peptides, exopolysaccharides, short-chain fatty acids (SCFAs), vitamins, peptidoglycan, lipopolysaccharides, cell surface proteins, and teichoic acids, and summarize their antimicrobial and immunostimulatory properties.

The present study aims to investigate and compare the immunomodulatory and antiviral properties of three heat-inactivated *Lactobacillus* strains, namely *L. paracasei* HA-108, *L. plantarum* HA-119, and *L. helveticus* HA-122, using an adult zebrafish model challenged with viral hemorrhagic septicemia virus (VHSV), as originally described by Novoa et al. (2006) [16]. By evaluating immune gene expression, gut and kidney histology, and survival outcomes in the zebrafish model, this study generates mechanistic and safety data that directly inform the development of postbiotic interventions aimed at enhancing immune health in companion animals.

## Materials and methods

The trial was performed at the Laboratory of Aquatic Animal Health, College of Veterinary Medicine, Chungnam National University (Daejeon, Republic of Korea). The protocol was approved by the Animal Ethics Committee of Chungnam National University (n°: 202203 A-CNU-019).

### Animals and husbandry

Wild-type adult zebrafish were bred in-house and maintained indoor within a zebrafish system equipped with mechanical and biological filtration, UV-disinfection, and aeration. The zebrafish experimental system used for the current study consisted of 15 tanks (20-L tanks, 22 fish per tank) connected to the previously described indoor system. Prior and during the trial; fish were kept under optimum water quality conditions (28 ± 1 °C, 14 h light: 10 h dark; dissolved oxygen > 5 mg/L, conductivity: 500–600 µS, total ammonia < 0.25 mg/L, nitrite < 0.3 mg/L), nitrate < 12.5 mg/L). All fish were fed with a commercial zebrafish diet prior to the start of the trial.

### Tested postbiotics and Preparation of experimental diets

A basal diet was formulated (Suppl. Table 1) using feed formulation software (Feedsoft^®^, version 10.1) in order to meet the known nutritional requirements of cyprinids [17]. Test diets were produced by cold-press extrusion based on the basal diet either not supplemented (Control) or supplemented. Three proprietary, commercially available, single-strain heat-treated *Lactobacillus* strains provided by Lallemand Health Solution (LHS, Blagnac, France), namely *L. paracasei* HA-108 (T1), *L. plantarum* HA-119 (T2) and *L. helveticus* HA-122 (T3), were tested at a concentration of 6 × 10^6^ cells/g of feed.

### Experimental design and sampling

Initially, the body weight of individual fish was measured, and fish were first allocated to large holding tanks before being distributed into the experimental tanks. Tanks were organized in a randomized block design to account for potential environmental gradients within the facility. Adult zebrafish (total 330) were divided into five experimental groups evaluated in triplicate tanks: two control groups received the Control diet and where either non-challenged with VHSV (negative Control; C1) or challenged (positive control; C2). The three other groups (T1, T2, and T3) were fed with one of the three experimental diets and challenged with VHSV. The experiment’s sampling strategy and challenge time frame are illustrated in Suppl. Figure 1.

The 31-day study comprised two distinct phases: a pre-challenge feeding phase (21 days) and a post-challenge phase (10 days). At the end of the feeding phase, 27 fish per group (9/tank) were randomly euthanized prior to sampling of the gut and kidney tissues to perform histomorphometry and immune response profiling using gene expression and immunoblotting. Remaining fish in the groups C2, T1, T2 and T3 (39 fish/group, 13/tank) were then challenged with VHSV and monitored for survival for 10 days post challenge (dpc). At 10 dpc, kidney tissues were collected (9 fish per group; 3/tank) for the evaluation of the virus copy number (VCN). Euthanasia was induced using 500 mg/L tricaine methanesulfonate (Sigma-Aldrich, St. Louis, MO, USA) by water immersion.

### VHSV challenge

For the virus challenge experiment, VHSV was propagated in a tissue culture flask (75 cm²) infecting the fathead minnow (FHM) cells according to Cho et al., 2019 [18] with minor modifications. In brief, cells were infected with a multiplicity of infection at 0.01 plaque-forming unit (pfu)/cell and kept at 20 °C. After a week, when an extensive cytopathic effect (CPE) had occurred, cell culture supernatant was centrifugated (3500 rpm at 4 °C for 15 min) and VHSV culture supernatant was harvested and stored at − 80 °C. Virus titration was determined by the 50% tissue culture infectivity dose (TCID50) experiment based on Spearman-Karber method [19]. For the challenge, 20 µL of VHSV suspension at a concentration of 10^8.8^ TCID50/mL (1.26 × 10^7^/fish) was injected intraperitoneally.

### Transcriptional analysis

Gut and kidney samples collected pre-challenge (day 21; 18 fish/group; 6/tank) were immediately snap-frozen in liquid nitrogen and stored at − 80 °C until conducting the transcriptional and protein expression analysis. Total RNA was extracted from gut and kidney tissues using TRIzol^®^ reagent (Invitrogen, MA, USA) according to the method described by Liyanage et al., (2024) [20]. RNA concentration was quantified using NanoDrop One (Thermo Scientific, MA, USA). Using total RNA (2.5 µg), cDNA was synthesized with the PrimeScript™ 1 st strand cDNA Synthesis Kit (TaKaRa, Tokyo, Japan) according to the manufacturer’s instructions. The primer description of selected immune genes is listed in Suppl. Table 2. Gene expression was analyzed by qRT-PCR (Thermal Cycler Dice^®^ Real-Time System; TaKaRa, Tokyo, Japan). The mRNA expressions were normalized to β-actin and expression-fold was determined by the 2^−ΔΔCt^ method [21]. Relative expression fold-changes were calculated by dividing the average expression- fold in the experimental diets (T1, T2 or T3) by that of the average expression of the negative control group (C1).

### Immunoblot analysis

Immunoblot analysis for selected immune functional proteins in the gut and kidney tissue (day 21; 9 fish/group, 3/tank) was performed according to Jayathilaka et al., (2024) [22]. Briefly, tissue samples were placed in ice-cold lysis buffer (300 µL; pH 7.6; ProEXTM CETi, TransLab Inc., Daejeon, Korea), homogenized for 1 min, centrifuged (12,000 × g at 4 °C for 10 min), and total protein amount was quantified by the Bradford method. Samples were then denatured (95 °C for 5 min with 2× Laemmli sample buffer; Sigma-Aldrich, St Louis, MO, USA) and a standard amount of protein (35 µg) was loaded into 10% sodium dodecyl sulfate-poly acrylamide gel (SDS-PAGE) and gel electrophoresis was performed (80 V, 30 min followed by 110 V, 1 h). The gel was then transblotted onto polyvinylidene difluoride (PVDF) membranes and blocked for 1 h with 5% bovine serum albumin (BSA). Membranes were incubated (overnight at 4 °C) with respective primary antibodies as listed in the Suppl. Table 3. Following incubation, membranes were washed three times with Tris-buffered saline containing 0.05% Tween 20 (TBST) and incubated (at 25 °C for 1 h) with respective goat anti-rabbit HRP (ABIN2690388) or mouse HRP (ABIN94386, both from Antibodies-online.com, Germany) secondary antibodies (1:20,000 in 5% BSA). The expressed proteins were detected using a chemiluminescence detection system (Fusion Solo S, Vilber, Lourmat, France). Glyceraldehyde-3-phosphate dehydrogenase protein (Gapdh) was considered as a housekeeping protein. Western blot images were acquired using different exposure times for each protein, which precludes reliable quantitative comparison of band intensities. Therefore, Western blot results are presented for qualitative assessment only, indicating the presence and relative changes of the target proteins rather than precise quantification.

### Tissue Preparation and histology analysis

For histological analysis, gut and kidney samples collected at pre-challenge (day 21; 9 fish/group, 3/tank) were preserved in 10% formalin and analyzed with a slightly modified method as described in our previous study [20, 23]. Briefly, all the specimens were dehydrated through an ascending graded series of alcohol and cleared with xylene using an automated tissue processor (Leica^®^ TP1020, Germany). Samples were then embedded in paraffin wax for transversal sectioning at 4 μm thickness (Leica^®^ RM2125 microtome, Germany), mounted and then stained. Specifically, the standard protocol of alcian blue (AB) and periodic acid-Schiff (PAS) staining (PAS stain kit, ab150680, CT, USA) was used to stain both acidic and neutral goblet cells in gut tissues while hematoxylin-eosin (H&E staining kit, ab245880, Abcam; CT, USA) staining was performed for the kidney tissues according to the manufacturer’s instructions prior to the cover slipping. Slides were observed and imaged at 40×, 100×, and 200× magnification under a light microscope connected to a digital camera (LEICA^®^ DCF450-C, Germany). Subsequently, images were analyzed using ImageJ 1.5 software [24] and goblet cells that were positive for AB-PAS staining were counted, and density and villi height were calculated [25].

### VHSV copy number analysis

For the determination of the VHSV copy number (VCN), kidney tissues collected at 10 dpc were snap-frozen in liquid nitrogen and stored at −80°C. Absolute quantification of VCN was performed using qRT-PCR as described in our previous study [20]. The total RNA of VHSV was isolated using the NucleoSpin^®^ RNA Virus kit (Macherey-Nagel, Duren, Germany) according to the manufacturer’s protocol. The VHSV nucleocapsid (N) gene was selected and it was amplified using specific primers (F: 5’-ATGACAAGCGCACTCAGAGAGA-3’ and R: 5’-TCAGTGGAATGAGTCGGAGTCTC-3’). PCR product was confirmed by 1.2% agarose gel electrophoresis and the amplified PCR product was ligated to pGEM^®^-T Easy Vector (Promega, Madison, WI, USA) following the procedure described by the manufacturer. Verification of the N gene construct was confirmed by sequencing. The N gene expression was quantified to plot a standard curve with the transformed plasmid concentration at the x-axis and threshold cycle (Ct) at the y-axis. Absolute quantification of VHSV in kidney tissues was determined by qRT-PCR using N gene-specific primers (F: 5’- ATCTGGAGGCAAAGTGCAAG − 3’ and R: 5’- CCATGAGGTTGTCGTTGTTG − 3’). The resulting Ct values were used with a standard curve to calculate the viral copy number (Suppl. Figure 2).

### Statistical analyses

GraphPad Prism software (version 5) for Windows (GraphPad Software Inc., San Diego, CA, USA) was used for all the data analyses. In the immune gene expression analysis study, data are presented as the mean ± standard error (SE) based on a minimum of three replicates. The mRNA expression data were analyzed by one-way analyses of variance (ANOVA) followed by Tukeys’s multiple comparison test. Statistical differences in zebrafish survival percentages between groups were determined using the Log-rank (Mantel-Cox) test. Results with p-values less than 0.05 were considered statistically significant.

## Results

### In vivo antiviral effects and viral load in postbiotics-supplemented zebrafish

To evaluate the in vivo antiviral efficacy of postbiotics, we monitored zebrafish survival rates before and after viral challenge across treatment groups, using survival as a proxy for overall health and immune defense.

**Fig. 1:**
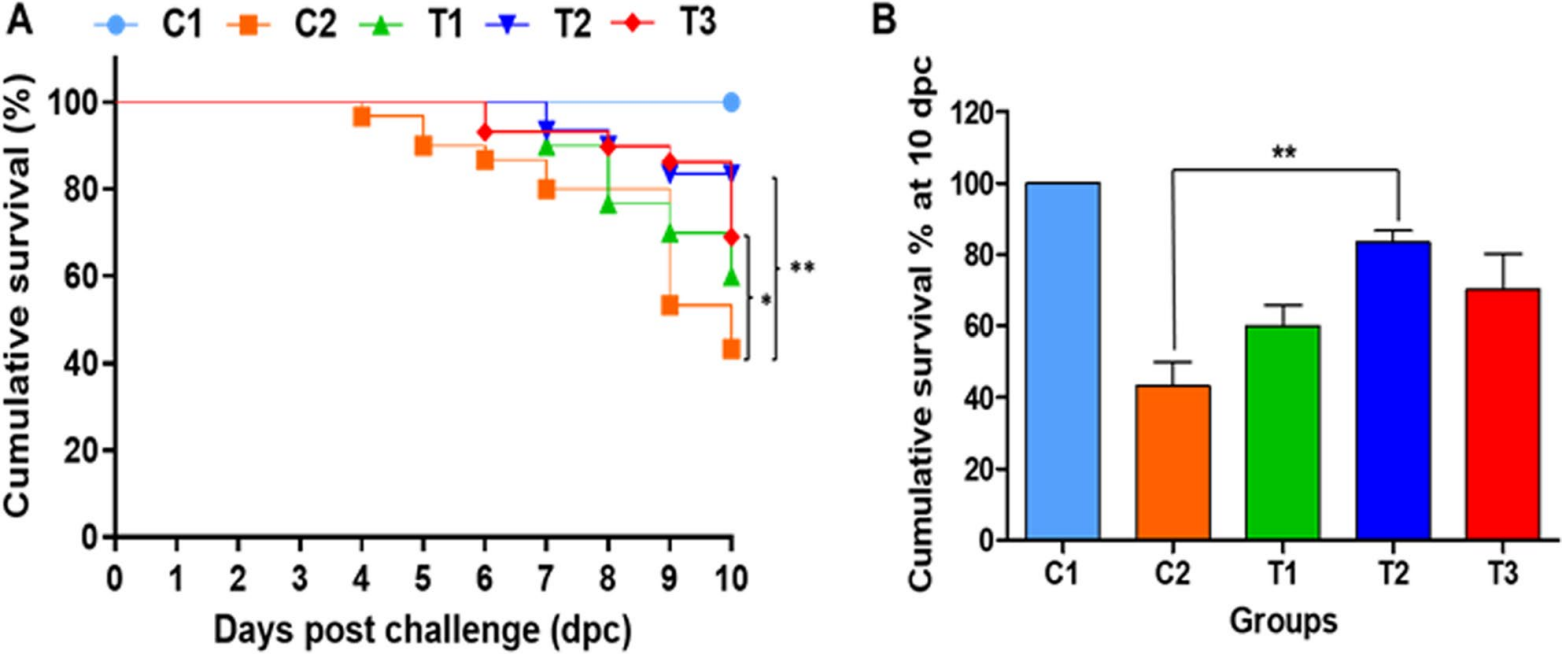
Effect of three bacterial-derived postbiotics on the survival and disease resistance of zebrafish against VHSV challenge. **A** Kaplan-Meier survival curve showing zebrafish survival over 10 days post challenge of VHSV. Statistical significance analysis of Log-rank (Mantel-cox) test is indicated by *p < 0.05 and **p < 0.01. **B** Cumulative survival percentage at 10 dpc. To assess statistical difference in cumulative survival percentage at 10 dpc, one-way analysis of variance (ANOVA) followed by Turkey’s multiple comparison test was performed. Statistical significance analysis is indicated by **p < 0.01. T1, T2, and T3 for *L. paracasei* HA-108, *L. plantarum* HA-119, and *L. helveticus* HA-122, respectively

**Fig. 2:**
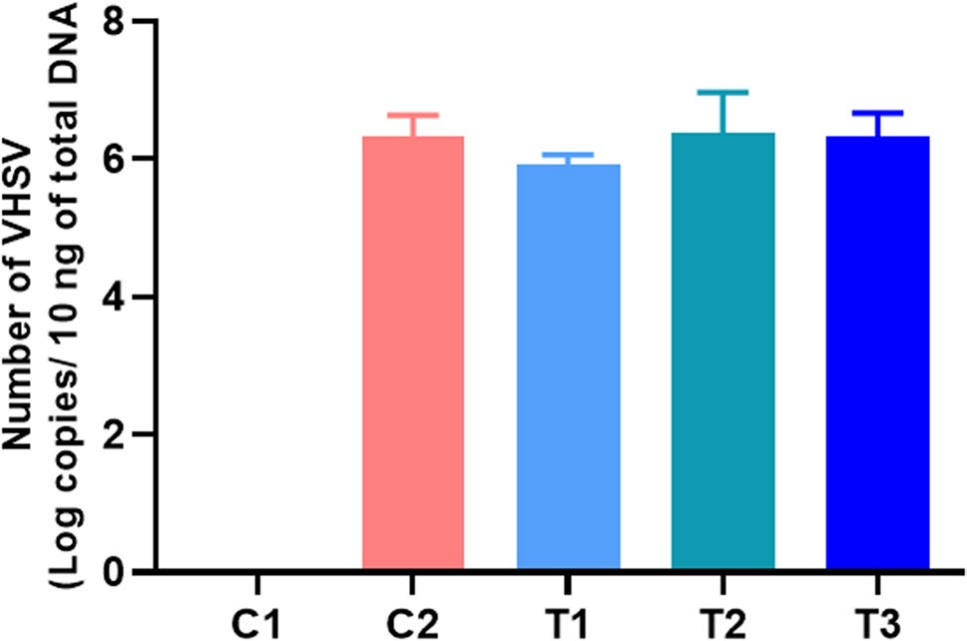
Effect of postbiotic supplementation on** v**iral clearance in zebrafish. Following VHSV challenge, kidney tissue was collected from surviving fish at 10 dpc. The VHSV copy number was quantified by amplifying the N gene fragment of VHSV using qRT-PCR, followed by calculation based on a standard curve-derived equation. The VHSV copy number was then normalized to 10 ng of template cDNA used in each PCR reaction (log copy number/10 ng of total DNA) as described by Kim et al., 2020. C1: unchallenged negative control group; C2: VHSV challenged control group. T1, T2, and T3: postbiotics-fed groups (*L. paracasei *HA-108,*L. plantarum *HA-119, and *L. helveticus* HA-122*) *subjected to VHSV challenge

Pre-challenge, no significant differences in survival were observed among groups, with survival rates of 95.6, 91.1, 92.2, 92.2, and 97.8% in the C1, C2, *L. paracasei* HA-108, *L. plantarum* HA-119, and *L. helveticus* HA-122 groups, respectively. Post-challenge, no mortality occurred in the negative control group (C1), while survival dropped 43.3% in the positive control (C2), confirming the effectiveness of the viral challenge model. In contrast, survival was improved in all postbiotic-treated groups: *L. paracasei* HA-108 (60.0%), *L. plantarum* HA-119 (83.3%), and *L. helveticus* HA-122 (70.0%) (Fig. 1A and B), with statistically significant improvements observed for *L. plantarum* HA-119 (*p* < 0.01) and *L. helveticus* HA-122 (*p* < 0.05). To further assess antiviral efficacy, viral load in kidney tissues was quantified by measuring VHSV copy number (VCN) in surviving fish at 10 dpc. As expected, no VCN was detected in the non-challenged C1 group. Among the challenged groups (Fig. 2), no statistically significant differences in VCN were observed, although a numerical reduction was noted in the *L. paracasei* HA-108 group (5.92 ± 0.15) compared to *L. plantarum* HA-119 (6.33 ± 0.32), *L. helveticus* HA-122 (6.26 ± 0.16), and challenged C2 group (6.30 ± 0.30 VHSV copies/10 ng of total DNA).

### Impact of postbiotics on gut structure and kidney health in zebrafish

**Fig. 3:**
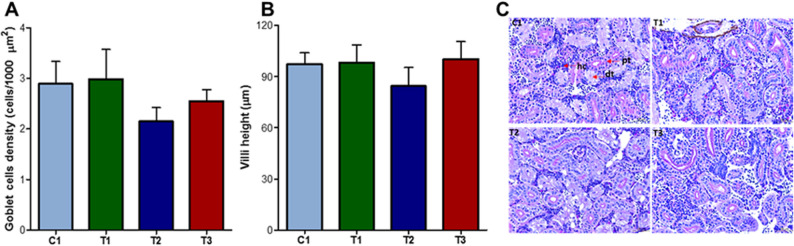
Histological analysis of gut and kidney tissues following dietary supplementation with three bacterial-derived postbiotics.**A** Goblet cell density and **B** Villi height in the gut were assessed, while **C **representative images of H&E-stained kidney tissues from control (C1) and postbiotic-fed groups (T1, T2, and T3 for *L. paracasei *HA-108, *L. plantarum *HA-119, and *L. helveticus* HA-122) are shown. Key structures, including the distal tubule (db), proximal tubule (pt), and hematopoietic cells (hc) are indicated by arrows. Scale bar: 25 µm

Across all experimental groups, the overall mean goblet cell density was 2.6 *±* 0.3 cells/1000 µm², and the average villus height was 94.5 *±* 10.6 μm. No statistically significant differences were observed between groups for either parameter, although numerical variations were noted and notably a reduction of both measurements with *L. plantarum* (Fig. 3A and B). Hematoxylin and eosin (H&E) stained histograms of the kidney tissue did not reveal any structural alterations between groups (Fig. 3C). Consistent structural elements of the zebrafish kidney including distal tubules, proximal tubules, and hematopoietic cells were evident across all groups. Inflammatory-related pathological symptoms, such as shrunken glomeruli and macrophage infiltration, were absent across all groups. Moreover, toxicity-related pathological signs and damage to the kidney, such as hemorrhages and cellular necrosis were not observed in any groups in this study.

### Modulation of gut and kidney immune responses by postbiotics

To assess the immunomodulatory effects of the three *Lactobacillus*-based postbiotics, we analyzed the expression of 16 immune-related genes in zebrafish gut and kidney tissues. These genes were selected for their roles in pattern recognition (e.g., toll like receptors: *tlr2*, *tlr4b*, *tlr5a*, *tlr5b*), inflammation (pro-anti-inflammatory cytokines: *il1β*,* tnfα*), chemotaxis (*ccl34a.4*, *cxcl18b*), antiviral defense (*infγ*, *infγ1r*, *Mx*, *cd8a*), antimicrobial activity (*defβ1*,* muc5.1*), and oxidative stress response (*cat*,* sod1*). Gene expression profiling was conducted at the end of the feeding phase (pre-challenge, day 21), and only genes with a fold change ≥ 1.5 were considered differentially expressed.

**Fig. 4:**
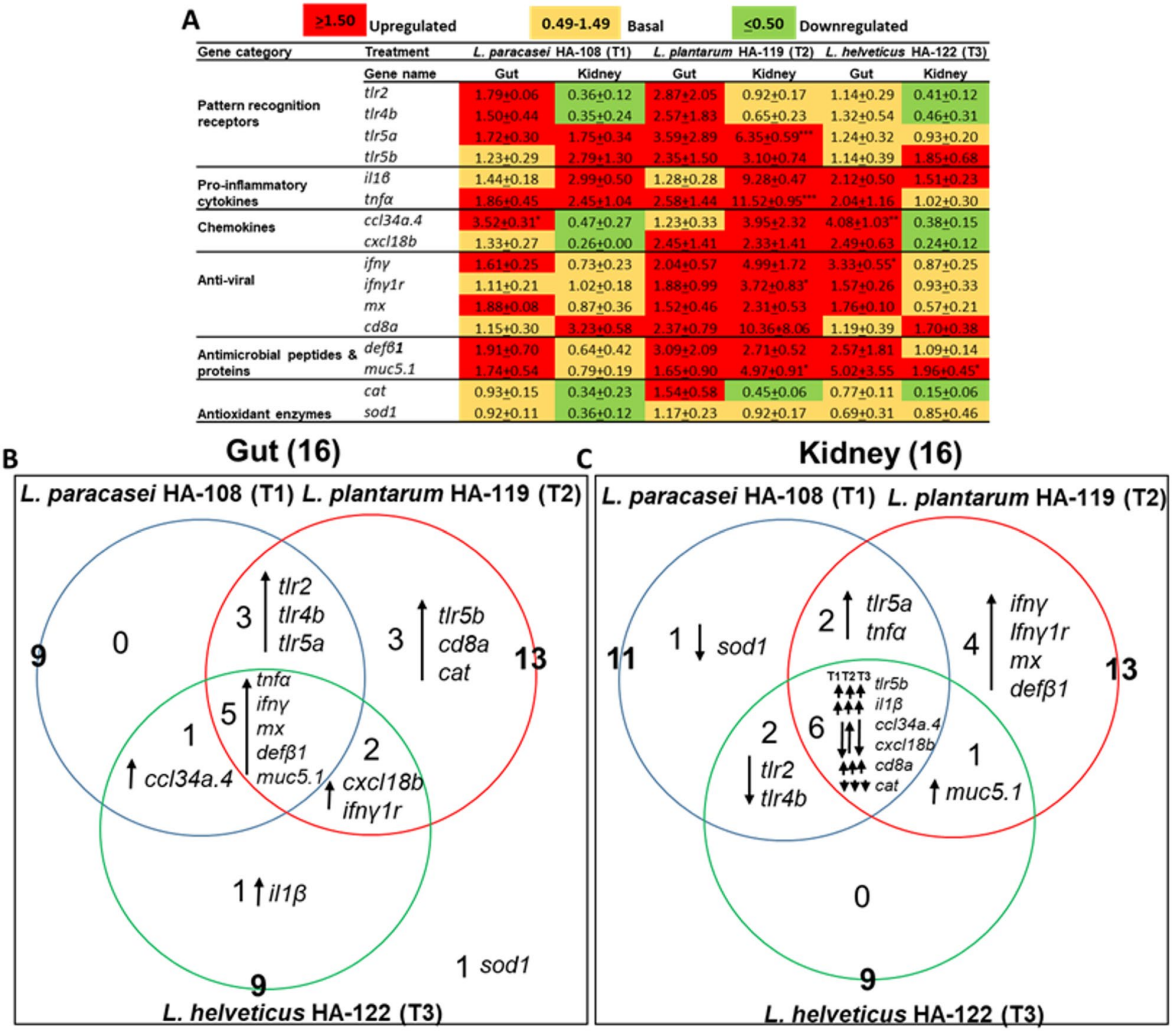
Differentially expressed (DE) immune genes in zebrafish gut and kidney following oral supplementation with bacterial-derived postbiotics. **A** Heatmap representation of mRNA expression levels in the zebrafish gut and kidney across three treatments. **B** Venn diagram showing tissue-specific DE immune genes in the gut and **C** kidney under the three treatments. The mRNA expression levels were normalized to β-actin analyzed using the 2^-ΔΔCT^ method. Relative fold-change in mRNA expression was calculated by dividing the average relative expression of each group by that of the control/vehicle diet (C1) at 21 dps. Expression scale: basal expression (yellow): 0.49-1.49 folds; upregulation (red): ≥ 1.5-fold, and downregulation (green): ≤ 0.50. To determine significant differences between the C1 and three treatments groups, one-way analysis of variance (ANOVA) followed by Turkey’s multiple comparison test was performed. Asterisk marks show the significant difference between C1 and treatment groups. *p < 0.05, **p < 0.01, and ***p< 0.01. T1, T2, and T3 for *L. paracasei *HA-108,*L. plantarum *HA-119, and *L. helveticus* HA-122, respectively

All three treatments induced distinct gene expression profiles in both gut and kidney tissues compared to the control group C1 (Fig. 4A). In the gut, the proportion of upregulated genes was 56% for *L. paracasei* HA-108, 81% for *L. plantarum* HA-119, and 56% for *L. helveticus* HA-122. In the kidney, the corresponding values were 31%, 75%, and 25%, respectively. Downregulated genes were observed only in the kidney, with 38% in the *L. paracasei* HA-108 group, 7% in the *L. plantarum* HA-119 group, and 32% in the *L. helveticus* HA-122 group.

Venn diagram analysis (Fig. 4B) revealed five genes commonly upregulated in the gut across all treatments (*tnfα*, *infγ*, *mx*, *defβ1*, *muc5.1*), and four genes consistently modulated in the kidney: *tlr5b*,* il1β*, and *cd8a* were upregulated, while *cat* was downregulated. Beyond these shared responses, each postbiotic induce distinct, strain-specific patterns of gene expression (Fig. 4A). *L. paracasei* HA-108 modulated genes across all functional categories in both tissues. *L. plantarum* HA-119 triggered the most extensive response, upregulating all genes except *sod1*. In contrast, *L. helveticus* HA-122 primarily affected genes related to inflammation, chemotaxis, antiviral, and antimicrobial defense, without modulating toll like receptor expression.

### Protein expression in gut and kidney tissues following postbiotic supplementation

**Fig. 5:**
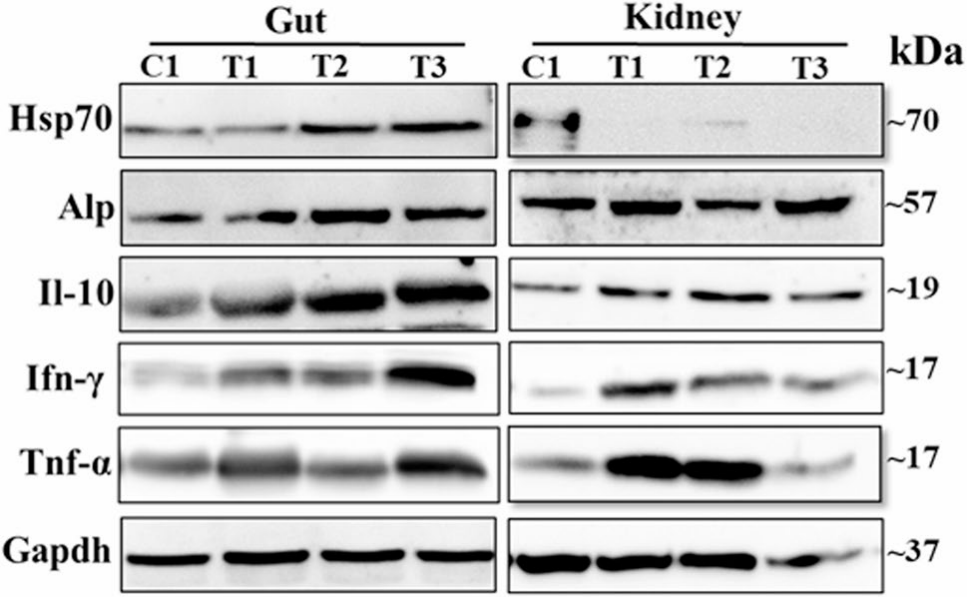
Immunoblotting analysis of selected immune functional proteins in the gut and kidney tissues of zebrafish following feeding bacterial-derived postbiotics. The image shows specific protein bands obtained using primary antibodies listed in Suppl. Table 2. T1, T2, and T3 for *L. paracasei*HA-108,*L. plantarum *HA-119, and *L. helveticus*HA-122, respectively

Immunoblotting analysis was performed to qualitatively assess the expression levels of five immune-related proteins (Tnf-α, Ifn-γ, Il10, Alp, and Hsp70) in gut and kidney tissues of zebrafish across treatment groups, compared to the control group C1 (Fig. 5, Suppl. Figure 3). Due to differences in exposure times during image acquisition, these results reflect the presence and relative changes of the target proteins rather than precise quantification. In the *L. paracasei* HA-108 group, four proteins (Tnf-α, Ifn-γ, Il10, and Alp) were overexpressed in both gut and kidney tissues, while Hsp70 was not overexpressed in either tissue. In the *L. plantarum* HA-119 group, Ifn-γ and Il10 were overexpressed in both tissues, Alp and Hsp70 were overexpressed in the gut, and Tnf-α was overexpressed only in the kidney. In the *L. helveticus* HA-122 group, Ifn-γ, Il10, and Alp were overexpressed in both gut and kidney tissues, while Tnf-α and Hsp70 were overexpressed only in the gut.

## Discussion

In the present study, we used a zebrafish model challenged with VHSV, as developed by Novoa et al. (2006) [16], to evaluate the effects of three inactivated *Lactobacillus* strains on gut health, immune response, and survival. The responses observed in the negative (non-challenged) and positive (challenged) control groups were consistent with expected outcomes, supporting the validity of the experimental conditions.

Toll-like receptors (TLRs) play a crucial role in recognizing pathogen-associated molecular patterns, initiating inflammatory responses, and bridging the innate and adaptive immune responses. At the initial stage of immune activation, pro-inflammatory cytokines such as TNF-α and IL-1β mediate the activation of other cytokines and chemokines, which modulate various immune responses such as phagocytic activity, leukocyte migration, macrophage proliferation in mammals and fish [26]. Chemokines like CCL34a.4 and CXCL18b are involved in recruiting immune cells and amplifying inflammatory responses during tissue repair, microbial infection, and antiviral defense. Zebrafish are widely used as a vertebrate model for studying host-microbiota interactions and dietary interventions, including postbiotics [27].

In this study, we examined the expression of 16 immune-related genes in gut and kidney tissues following postbiotic supplementation. The three postbiotics induced differential gene expression in a tissue-specific manner. In the gut, 56.3%, 81.3%, and 68.8% genes were differentially expressed in the *L. paracasei* HA-108, *L. plantarum* HA-119, and *L. helveticus* HA-122 groups, respectively; in the kidney, the corresponding values were 68.8%, 81.3%, and 56.3%. These differences suggest that each of the three postbiotics may contain distinct metabolites or surface molecules that interact with host tissues to activate specific immune pathways.

Importantly, we observed a common signature for the three selected postbiotics, characterized by an upregulation of *tnfα*, *ifnγ*, *mx*, *defβ1*, and *muc5.1* in the gut, and *tlr5b*, *il1β*, and *cd8a* in the kidney. These genes encode proteins central to antiviral defense and mucosal barrier function. *Defensin* are antimicrobial peptides involved in the direct killing of pathogens [28, 29]; *mucins* regulate growth, virulence, quorum sensing, and pathogen adhesion [30]; *IFNγ* modulates macrophage and T cell activation [31]; and *Mx* inhibits viral replication. The coordinated upregulation of these genes and their corresponding proteins, confirmed by immunoblotting, supports a model in which postbiotics enhance both innate and adaptive immune responses, thereby conferring increased resilience to viral challenge.

We detected some species-dependent effects, as the upregulated *ccl34a.4* (with *L. paracasei* HA-108 and *L. helveticus* HA-122) and *cxcl18b* (with *L. plantarum* HA-119 and *L. helveticus* HA-122) indicates possible activation of immune cell recruitment to eliminate the pathogens. Comparative analysis revealed 13 genes (81%) were more than 1.5-fold overexpressed by *L. plantarum* postbiotic treatment, including *tlr5b*, *cd8a*, and *cat* in the gut. CD8 is a cell surface glycoprotein associated with cytotoxic T cells, which play an important role in killing or eliminating virus-infected cells. Rawling et al. (2023) [2] described higher CD8 + T cells with the *L. plantarum* HA-119 postbiotic diet in healthy zebrafish and suggested that it could be associated with induced *ifnγ* expression. The antioxidant enzyme catalase converts hydrogen peroxide, thereby reducing cellular oxidative stress. SWFC-fed common carp (*C. carpio*) had upregulated catalase expression [27] and upregulated catalase by *L. plantarum* HA-119 postbiotic treatment in the gut indicates its important role on maintaining the redox balance and cellular homeostasis. Overall, these findings suggest that *L. plantarum* HA-119 postbiotic treatment could modulate immune surveillance, promote cytotoxic T-cell activity, and enhance antioxidant defenses within the gastrointestinal tract.

In our study, dietary supplementation of zebrafish with *L. plantarum* HA-119 postbiotic induced AMPs, *defβ1* and antiviral genes (*ifnγ*, *ifnγ1r*, and *mx*) expression in the kidney, suggesting activation of antiviral pathways. Further, *tlr5a*, *tnfα*, and *il1β* were significantly expressed compared to the control diet and the *L. helveticus* HA-122 treatment, suggesting its possible role in inflammatory responses. Although, the kidney showed differentially expressed genes with a complex immunomodulatory response, a numerically higher number of genes were upregulated in the gut than the kidney, which may lead to the conclusion that the primary effects of the postbiotics used in this study are based on the reinforcement of the gut barrier function.

These findings are consistent with previous studies in aquatic and mammalian models. For example, Rawling et al., (2023) [2] reported that the same heat-inactivated *L. helveticus* HA-122 and *L. plantarum* HA-119 induced the expression of *tnfα*, *il1β*, *ifnγ*, *il22*, *il17a*, and *tgfβ* in healthy zebrafish. The induction of ifnγ has also previously been reported with commercial formulations containing live *L. helveticus* R0052 [32] and with *L. rhamnosus* GG in infants with cow’s milk allergy [33].

*Lactobacillus*-based postbiotics contain diverse surface components that can work together to strengthen the gut barrier and reduce inflammation. The peptidoglycan of *L. casei* YIT 9029, *L. johnsonii* JCM 2012, and *L. plantarum* ATCC 14,917 has been reported to suppress IL-12 production via Toll-like receptor 2 (TLR2), which is associated with autoimmune and inflammatory bowel diseases [34]. Lipoteichoic acid from *L. plantarum* also demonstrated anti-inflammatory responses in porcine intestinal epithelial cells [35]. Studies have revealed that exopolysaccharides (EPS) derived from several species of *Lactobacillus* have the capacity to modulate systemic and mucosal immune responses, providing direct health-promoting benefits [36, 37]. EPS produced by *L. plantarum* N14 was able to decrease the production of pro-inflammatory cytokines (IL-6, IL-8, and MCP-1) in porcine intestinal epithelial cells in response to enterotoxigenic *Escherichia coli* (ETEC) challenge [36]. The anti-inflammatory functions and effects of the surface components are species- or strain-specific. For instance, it has been shown that most immunomodulatory properties induced by *L. plantarum* teichoic acids were dependent on D-alanylation [38]. Notably, strain- or species-specific modifications of the conserved peptidoglycan polymers, including amidation, acetylation, and glycosylation, can lead to specific immunomodulatory capacities, contributing to the strain-specificity. Therefore, characterizing the effects of each strain of postbiotics on gut function is essential for strategic implementation and improving personalized health concepts.

Key immune functional proteins were tested to further confirm the transcriptional results and to evaluate the effects of each postbiotic at the protein level. Immunoblot results display differently induced proteins in three postbiotic fed zebrafish although expression patterns are different in the gut and kidney. This tissue-specific expression may reflect differences in the immunocompetence of the gut and kidney, as well as variations in postbiotic composition. A study conducted by Jensen et al., (2017) [39] described that cell wall components of probiotic *Bacillus coagulans* GBI-30 stimulated the anti-inflammatory cytokine IL-10 and decreased pro-inflammatory TNF-α and IFN-γ together suggesting it may balance Th1/Th2 immune response. Up-regulation of IL-10 level agreed with the present results although initial immune stimulation could be the root cause that led to the production of anti-inflammatory IL-10. Therefore, it could be hypothesized that the postbiotics used in this study activated both innate and adaptive immune responses leading to an initial inflammatory state.

Histopathological evaluation of the gut and kidney provides valuable insights into the overall health status of fish and helps distinguish between immune modulation induced by treatment or that resulting from pathological conditions [40–42]. Previous studies have shown that dietary supplementation with postbiotics in aquatic species such as common carp [27], oriental river prawn [43], and Pacific white shrimp [44] can enhance intestinal morphology, including increased microvilli length and improved interaction between epithelial cells and the basement membrane, thereby supporting efficient nutrient absorption. In our study, postbiotic supplementation did not induce any apparent alterations in gut histo-morphological structure, although only goblet cell density and villus height were assessed. The absence of structural changes suggests that the treatments were well tolerated and did not exert harmful effects on the intestinal architecture. Similarly, kidney histology revealed no pathological signs such as glomerular shrinkage, immune cell infiltration, necrosis, or inflammatory lesions, further supporting the non-toxic nature of the postbiotics at the administered dose [40, 45–47].

Several studies have demonstrated that dietary supplementation with postbiotics can enhance resistance to pathogenic infections in aquatic species. For instance, white shrimp (*Litopenaeus vannamei*) fed with postbiotics *B. licheniformis* BCR 4–3 and *Vibrio parahaemolyticus* IPNGS16 were more resilient against *V. parahaemolyticus* [48]. Similarly, Perez-Sanchez et al. (2020) [9] reported that postbiotics from lactic acid bacteria (*Lactobacillus* and *Leuconostoc* spp.) prevented the development of *Lactococcus garvieae* infection in rainbow trout (*Oncorhynchus mykiss*), likely through modulation of the intestinal microbiota. In vitro, Vilhelmova-Ilieva et al. (2023) [49] showed that post metabolites from *L. plantarum* L3 and *L. gasseri* VS (human origin) exerted antiviral effects against koi herpesvirus in common carp (*C. carpio*) brain cells. In our study, although VCN were similar across groups, fish fed with *L. plantarum* HA-119 and *L. helveticus* HA-122 exhibited significantly higher survival rates following VHSV challenge compared to the control group C2 fed a basal diet (*p* < 0.001 and *p* = 0.018, respectively). While the difference in survival for the *L. paracasei* HA-108 group was not statistically significant, a numerically lower VHSV copy number was observed, suggesting a potential protective trend.

While the zebrafish model offers significant advantages for mechanistic studies of mucosal immunity and high-throughput screening, certain limitations must be acknowledged when extrapolating findings to companion animals such as dogs and cats. Differences in gut physiology, microbiota composition, immune system complexity, dietary requirements, and metabolic rates may affect the translatability of results between fish and mammals. Thus, although our study provides valuable mechanistic insights, further validation in target companion animal species is warranted to confirm efficacy and to optimize dosing strategies. Notably, our study evaluated postbiotic effects at a single dose, reflecting a broader gap in the field. Dose-response relationships for postbiotics remain largely unexplored in both aquaculture and companion animal research. Recent reviews have highlighted the lack of standardized protocols and species-specific dose-response studies for postbiotics [13, 14]. Future research should therefore include systematic dose-response experiments to determine the optimal concentrations required for efficacy and safety in target species.

## Conclusions

This study demonstrated the strain-specific immunomodulatory properties of *L. paracasei* HA-108, *L. plantarum* HA-119, and *L. helveticus* HA-122 using the zebrafish model. Enhanced resilience to viral challenge was observed, particularly in groups fed *L. plantarum* HA-119 and *L. helveticus* HA-122, and was associated with the upregulation of key immune markers, including cytokines, chemokines, and antimicrobial peptides. These immune pathways are conserved across vertebrates and function similarly in companion animals, suggesting that postbiotics may offer comparable immunomodulatory benefits in pets, potentially enhancing gut mucosal defense, viral resistance, and overall health. Further research is warranted to evaluate the longer-term effects of postbiotic supplementation, assess their efficacy in target species such as dogs and cats, and optimize practical aspects such as dosage and administration. Such studies will be essential for establishing postbiotics as reliable and functional dietary ingredients in companion animal nutrition.

## Supplementary Information

Supplementary Material 1.

Supplementary Material 2.

Supplementary Material 3.

Supplementary Material 4.

## Data Availability

The datasets used and/or analysed during the current study are available from the corresponding author on reasonable request.
