# Supplementary figures and images for: Identification of immunomodulating properties of postbiotics from lactobacilli using the zebrafish (Danio rerio) model
Source: BMC Vet Res. 2025 Nov 28;22:1. doi: 10.1186/s12917-025-05159-z (PMC12764115; doi:10.1186/s12917-025-05159-z)

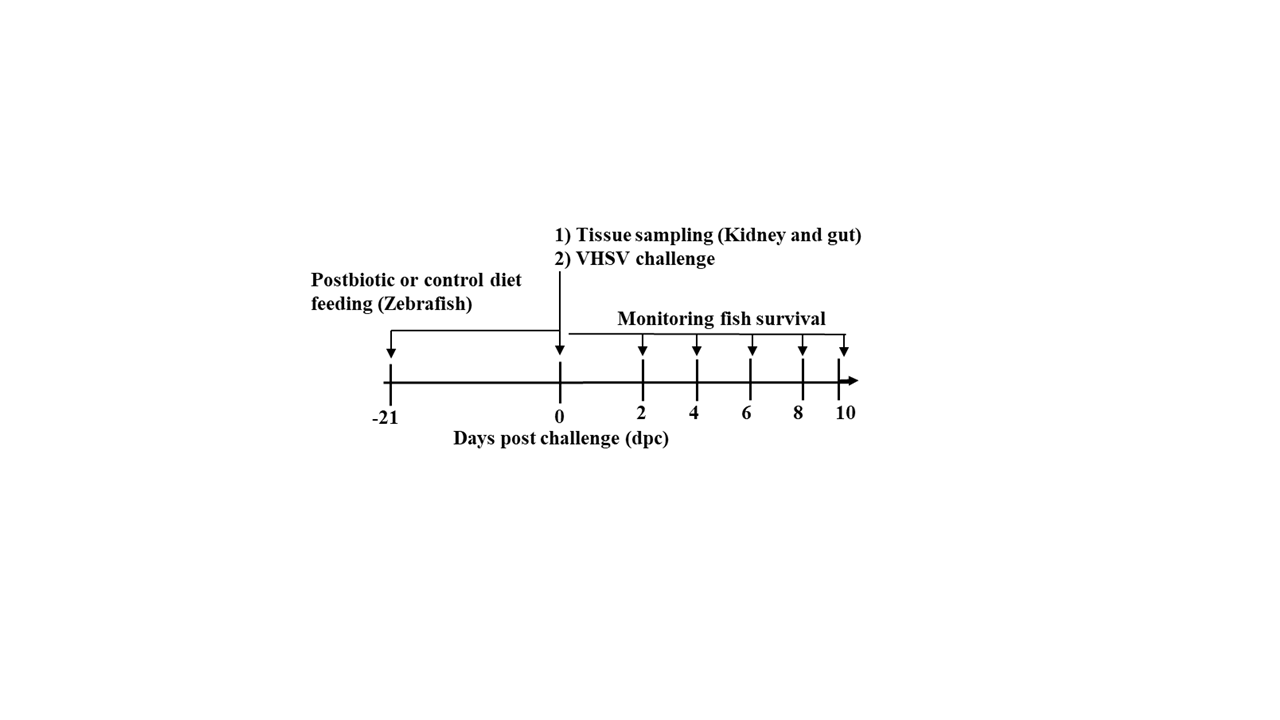

Supplement: Supplementary file 1 — Supplementary Material 1. [file 12917_2025_5159_MOESM1_ESM.tif]

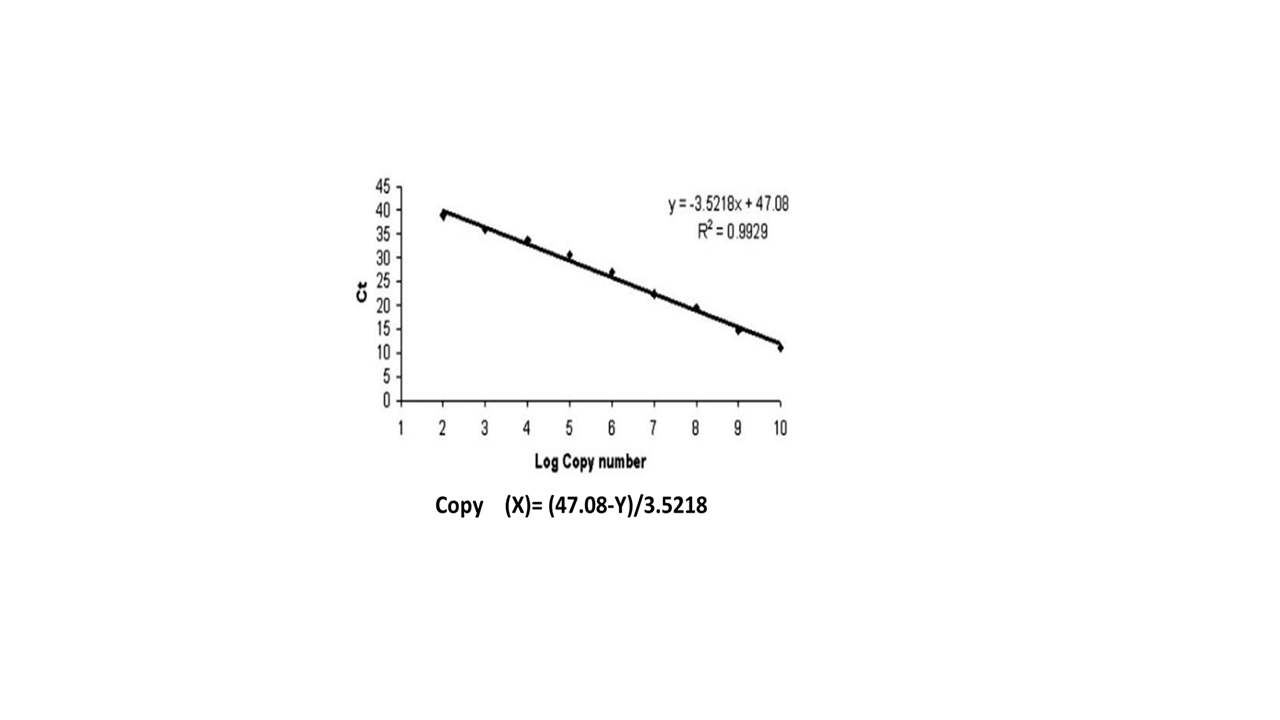

Supplement: Supplementary file 2 — Supplementary Material 2. [file 12917_2025_5159_MOESM2_ESM.tif]

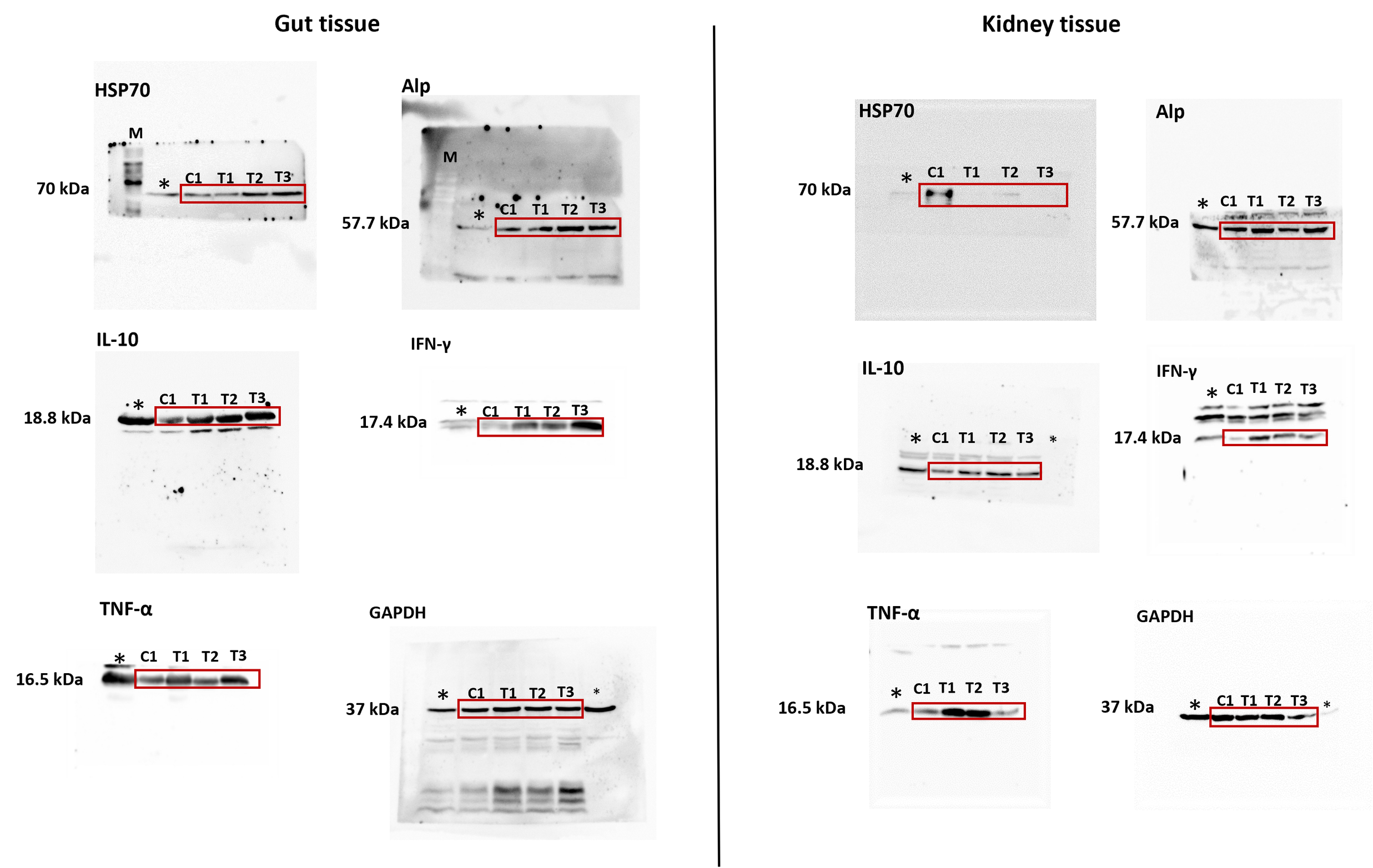

Supplement: Supplementary file 4 — Supplementary Material 4. [file 12917_2025_5159_MOESM4_ESM.tif]
