# Supplementary material for: Identification of immunomodulating properties of postbiotics from lactobacilli using the zebrafish (Danio rerio) model
Source: BMC Vet Res. 2025 Nov 28;22:1. doi: 10.1186/s12917-025-05159-z (PMC12764115; doi:10.1186/s12917-025-05159-z)
Supplement: Supplementary file 3 — Supplementary Material 3. [file 12917_2025_5159_MOESM3_ESM.docx]

**Additional File 1**

**Supplementary Table 1.** Feed formulation for experimental fish diet.

| Ingredients | % inclusion | per g/kg |
| --- | --- | --- |
| Wheat meal | 32.7 | 326.85 |
| Sunflower meal | 5.0 | 50.00 |
| Soybean meal | 20.0 | 200.00 |
| SPC60 | 32.4 | 323.89 |
| Rapeseed oil | 6.6 | 65.79 |
| Vitamin premix | 1.0 | 10.00 |
| Lysine HCL | 0.1 | 1.47 |
| DL methionine | 1.1 | 11.00 |
| Gelatin | 1.0 | 10.00 |

**Supplementary Table 2.** List of primers used for the qRT-PCR analysis.

| **Gene name** | **TM (°C)** | **Primer name** | **Primer sequences (5′-3′)** | **Accession number** |
| --- | --- | --- | --- | --- |
| Toll like receptor 2 (*tlr2*) | 58 | *tlr2-F* | TCTCCGTCTTGGTTTCAC | NM_212812.1 |
|  |  | *tlr2-R* | GGTCCCACAGTTGAGTATG |  |
| Toll like receptor 4 (*tlr4*) | 58 | *tlr4-R* | GGAATAATGGGCAGCCGTAAG | AY388400.1 |
|  |  | *tlr4-R* | AGCGACACCAGGAACTATCAATG |  |
| Toll like receptor 5a (*tlr5a*) | 58 | *tlr5a-F* | ACTCCGCTGTTGCTTTGA | AY389449.1 |
|  |  | *tlr5a-R* | GTTTAGACACCACGCAAATGG |  |
| Toll like receptor 5b (*tlr5b*) | 58 | *tlr5b-F* | GAAACATTCACCCTGGCACA | BC163185.1 |
|  |  | *tlr5b-R* | CTACAACCAGCACCACCAGAATG |  |
| Tumor necrosis factor alpha (*tnfα*) | 58 | *tnfα-F* | AGAAGGAGAGTTGCCTTTACCGCT | AY427649 |
|  |  | *tnfα-R* | AACACCCTCCATACACCCGACTTT |  |
| Interleukin 1 beta (*il1β*) | 58 | *il1b-F* | TCAAACCCCAATCCACAGAG | AY340959.1 |
|  |  | *il1b-R* | TCACTTCACGCTCTTGGATG |  |
| Chemokine (C-C motif) ligand 34a (*ccl34a.4*) | 55 | *ccl34a.4-F* | TGCAGCTCAACCAGAAGATG | BC162421.1 |
|  |  | *ccl34a.4-R* | CTTTGACGCATGGAGGATTT |  |
| Chemokine CXCL-C1C (*cxcl18b*) | 55 | *cxcl18b-F* | CTGCTGCTCGCGGTAGTTTA | NM_001115060 |
|  |  | *cxcl18b -R* | TCAACTTTGTCGCAGTTTGG |  |
| Interferon gamma 1(*ifnγ*) | 56 | *ifnγ-F* | ATGATTGCGCAACACATGAT | AB158361 |
|  |  | *ifnγ-R* | ATCTTTCAGGATTCGCAGGA |  |
| Interferon gamma related 1(*ifnγ1r*) | 58 | *ifnγ1-F* | AAATGGTGCTACTCTGTGGAC | NM_001020793 |
|  |  | *ifnγ1-R* | TGGACCGTCGAAATCTGTATG |  |
| Mx protein (*mx*) | 62 | *mx-F* | AGTCTGCAGATCACCTCTGCCAAT | NM_182942 |
|  |  | *mx-R* | ACCACGACCAGGTTGATTGTCTCT |  |
| Cluster of differentiation 8a (*cd8a*) | 56 | *cd8a-F* | AAGAGCATAGCACCGTAG | BC162235.1 |
|  |  | *cd8a-R* | GACTTCCGTCTGCTTTGCG |  |
| Beta defensin like 1 (defβ1) | 62 | *defβ1-F* | TGTGCAAGTCTCAGTGGTGTTTGC | NM_001081553 |
|  |  | *defβ1-R* | TTTGCCACAGCCTAATGGTCCGAA |  |
| Mucin 5.1 (*muc5.1*) | 56 | *muc5.1-F* | TGGCAACTTGGCTGATGATA | XM_009297795.1 |
|  |  | *muc5.1-R* | TCGTCACACGGACCAGTAGA |  |
| Catalase (*cat*) | 58 | *cat-F* | CCAAGGTCTGGTCCCATAAAG | NM_130912.2 |
|  |  | *cat-R* | GCTCAACCTCCGCGAAATA |  |
| Superoxide dismutase 1 (*sod1*) | 58 | *sod1-F* | AGGTGACTGGTGAAATTACTGG | NM_131294.1 |
|  |  | *sod1-R* | GTCTCACACTATCGGTTGGC |  |
| β- actin | 58 | *β-actin-F* | AATCTTGCGGTATCCACGAGACCA | AF025305 |
|  |  | *β-actin-R* | TCTCCTTCTGCATCCTGTCAGCAA |  |
|  |  |  |  |  |

**Supplementary Table 3.** List of antibodies used for immunoblotting analysis.

| **Primary antibody** | **Antibody dilution** | **Catalog number**  **and manufacturer** | **Secondary**  **antibody** | **Antibody dilution** | **Catalog number**  **and manufacturer** |
| --- | --- | --- | --- | --- | --- |
| TNF-α | 1:1000 | KP1540Z-100 (Kingfisher Biotech, Inc, MI, USA) | Anti-rabbit Ig-G-HRP | 1:3000 | #7074 (Cell signalling Technology, MA, USA) |
| IFN-ϒ | 1:1000 | KP1233Z-100 (Kingfisher Biotech, Inc, MI, USA) | Anti-rabbit Ig-G-HRP | 1:3000 | #7074 (Cell signalling Technology, MA, USA) |
| IL-10 | 1:1000 | KP1267Z-100 (Kingfisher Biotech, Inc, MI, USA) | Anti-rabbit Ig-G-HRP | 1:3000 | #7074 (Cell signalling Technology, MA, USA) |
| ALP | 1:1000 | GTX112100 (GeneTex, Quebec, Canada) | Anti-rabbit Ig-G-HRP | 1:3000 | #7074 (Cell signalling Technology, MA, USA) |
| Hsp70 | 1:1000 | #4872 (Cell signalling Technology, MA, USA) | Anti-rabbit Ig-G-HRP | 1:3000 | #7074 (Cell signalling Technology, MA, USA) |
| GAPDH | 1:1000 | Sc-365062 (Santa Cruz Biology Inc, OR, USA) | Anti-mouse Ig-G-HRP | 1:3000 | GTX2113111-01 (GeneTex, Quebec, Canada) |

**
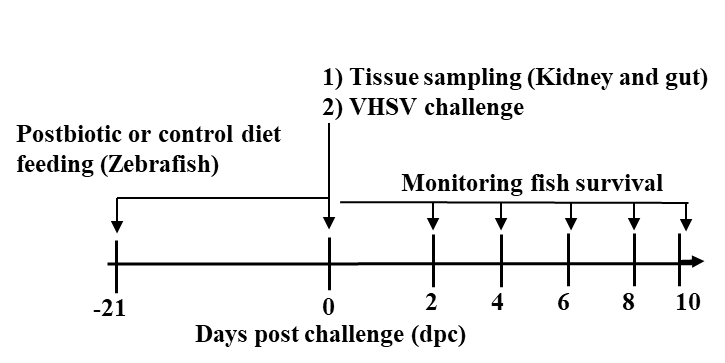
**

**Supplementary Figure 1**. Schematic representation of experimental layout and methodology.

**
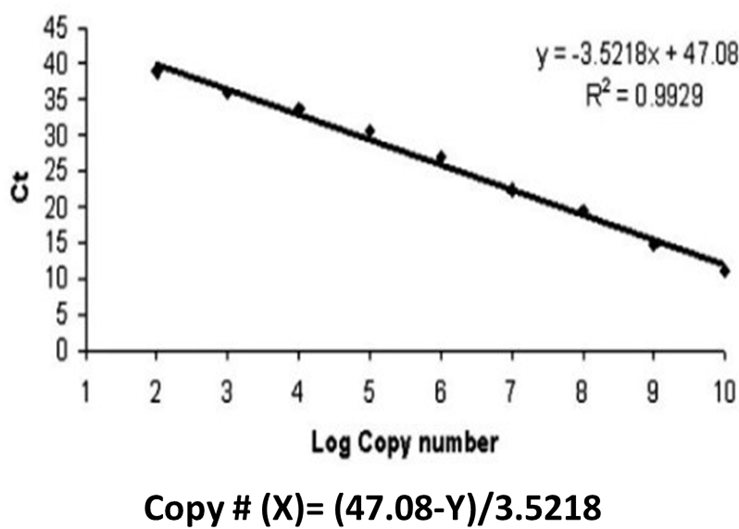
**

**Supplementary Figure 2.** Standard curve for VHSV copy number calculation. A standard curve is created using serial dilutions of a known concentration of a VHSV plasmid by analyzing the N gene expression (by qRT-PCR) and threshold cycle (Ct).


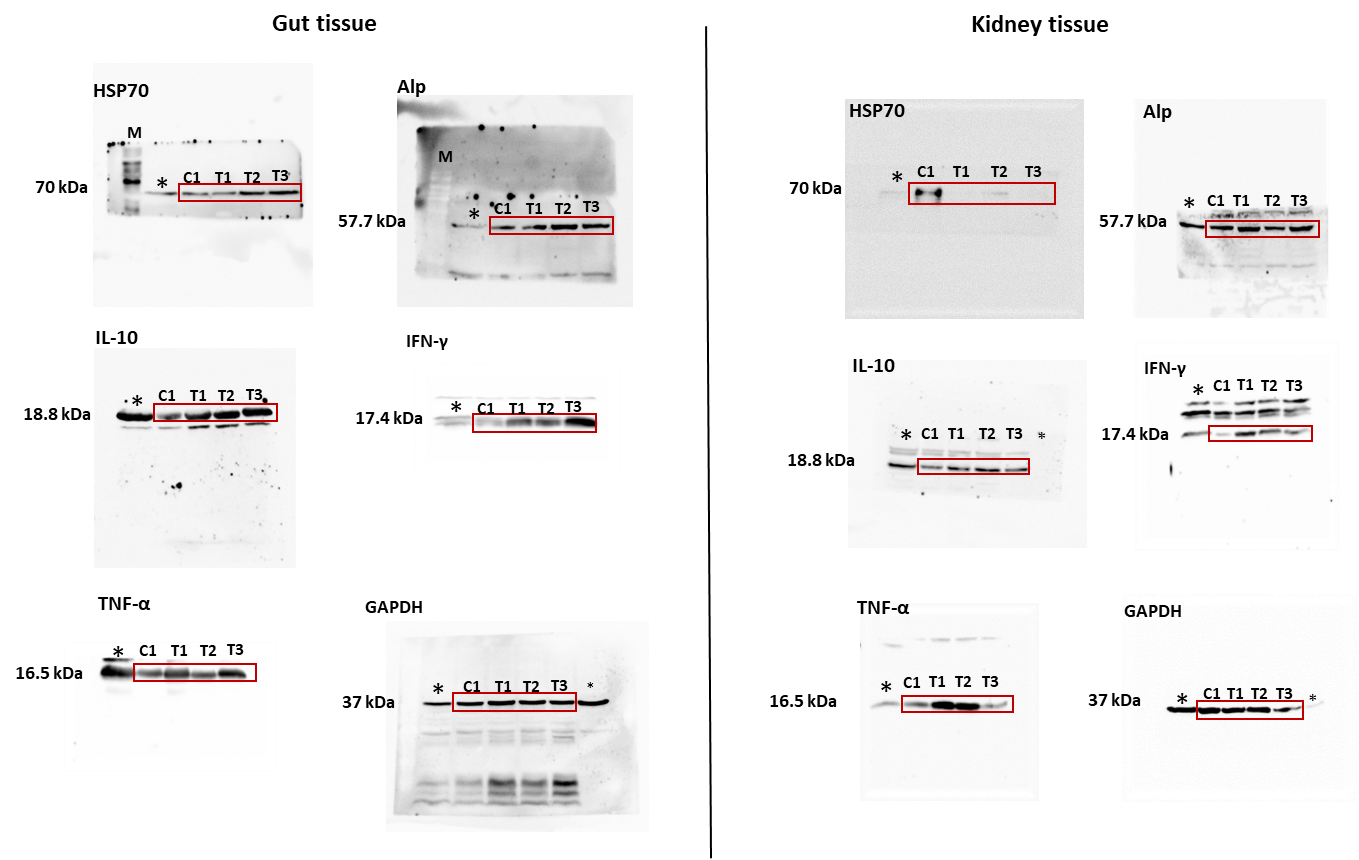


**Supplementary Figure 3.** Original gel blots images correspond to the western blot data presented in Figure 5. The relevant lanes with corresponding samples included Figure 5 are indicated as C1, T1, T2, and T3. To avoid the inclusion of non-specific signals, the gel blots were sectioned to retain only the molecular weight regions of protein interest prior to antibody incubation. This approach excluded areas displaying non-specific band detection. Furthermore, to minimize the background noise during image acquisition, regions showing non-specific bands were masked using a black board (Ifnγ and TNFα). This procedure ensured that only the specific protein bands of interest were visualized and presented. Lane marked in asterisk (*) represents samples not relevant to the data shown in Figure 5.
